# 7-Fluoro-2-(prop-2-en-1-ylsulfan­yl)-3-(1*H*-1,2,4-triazol-1-yl)-4*H*-thio­chromen-4-one

**DOI:** 10.1107/S1600536811022665

**Published:** 2011-06-22

**Authors:** Dong-liang Liu, Tao Xiao, Yang Li, Guang-yan Yu, Chen Li

**Affiliations:** a Department of Applied Chemistry, College of Science, Nanjing University of Technology, Nanjing 210009, People’s Republic of China

## Abstract

The asymmetric unit of the title compound, C_14_H_10_FN_3_OS_2_, contains two independent mol­ecules which differ in the relative orientations of the triazole and allyl­sulfanyl groups with respect to the planar thio­chromen-4-one frameworks. The N—N—C—C torsion angles are 128.2 (5) and −120.9 (5)°, while the C—S—C—S torsion angles are −17.4 (4) and 16.4 (4)°. In the crystal, inter­molecular C—H⋯O and C—H⋯N hydrogen bonds link the mol­ecules in a stacked arrangement along the *a* axis.

## Related literature

For related compounds containing a 4*H*-thio­chromen-4-one fragment, see: Adams *et al.* (1991 ▶); Nakazumi *et al.* (1992 ▶); Weiss *et al.* (2008 ▶); Li *et al.* (2010*a* ▶,*b* ▶); Xiao *et al.* (2010 ▶). For bond-length data, see: Allen *et al.* (1987 ▶).
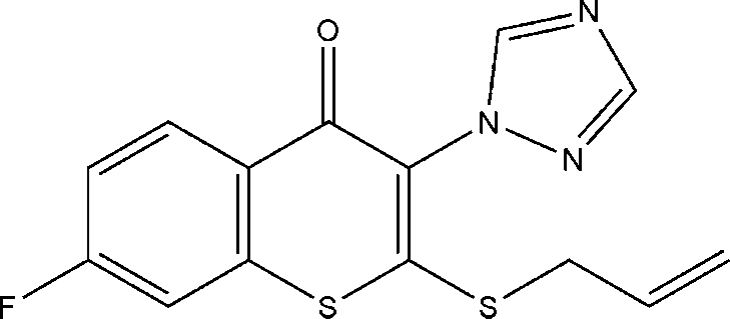


## Experimental

### 

#### Crystal data

C_14_H_10_FN_3_OS_2_
*M* *_r_* = 319.39
Triclinic, 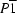

*a* = 8.1730 (16) Å
*b* = 11.646 (2) Å
*c* = 15.124 (3) Å
α = 82.43 (3)°
β = 83.98 (3)°
γ = 80.14 (3)°
*V* = 1400.9 (5) Å^3^
*Z* = 4
Mo *K*α radiation
μ = 0.39 mm^−1^
*T* = 293 K
0.20 × 0.10 × 0.10 mm

#### Data collection

Enraf–Nonius CAD-4 diffractometer
Absorption correction: ψ scan (North *et al.*, 1968 ▶) *T*_min_ = 0.926, *T*_max_ = 0.962
5541 measured reflections
5149 independent reflections
3087 reflections with *I* > 2σ(*I*)
*R*_int_ = 0.037
3 standard reflections every 200 reflections  intensity decay: 1%

#### Refinement

*R*[*F* ^2^ > 2σ(*F* ^2^)] = 0.069
*wR*(*F* ^2^) = 0.176
*S* = 1.01
5149 reflections
379 parameters
1 restraint
H-atom parameters constrained
Δρ_max_ = 0.52 e Å^−3^
Δρ_min_ = −0.40 e Å^−3^

### 

Data collection: *CAD-4 Software* (Enraf–Nonius, 1985) ▶; cell refinement: *CAD-4 Software* ▶; data reduction: *XCAD4* (Harms & Wocadlo, 1995 ▶); program(s) used to solve structure: *SHELXS97* (Sheldrick, 2008 ▶); program(s) used to refine structure: *SHELXL97* (Sheldrick, 2008 ▶); molecular graphics: *PLATON* (Spek, 2009 ▶); software used to prepare material for publication: *SHELXTL* (Sheldrick, 2008 ▶).

## Supplementary Material

Crystal structure: contains datablock(s) I, global. DOI: 10.1107/S1600536811022665/zq2103sup1.cif

Structure factors: contains datablock(s) I. DOI: 10.1107/S1600536811022665/zq2103Isup2.hkl

Supplementary material file. DOI: 10.1107/S1600536811022665/zq2103Isup3.cml

Additional supplementary materials:  crystallographic information; 3D view; checkCIF report

## Figures and Tables

**Table 1:** Hydrogen-bond geometry (Å, °)

*D*—H⋯*A*	*D*—H	H⋯*A*	*D*⋯*A*	*D*—H⋯*A*
C1—H1*A*⋯O2^i^	0.93	2.50	3.328 (5)	149
C4—H4*A*⋯N5^ii^	0.93	2.61	3.397 (7)	143
C15—H15*A*⋯O1	0.93	2.56	3.372 (6)	147
C18—H18*A*⋯N2^iii^	0.93	2.48	3.355 (7)	157

Symmetry codes: (i) 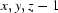; (ii) 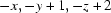; (iii) 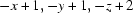.

**Fig. 1.**
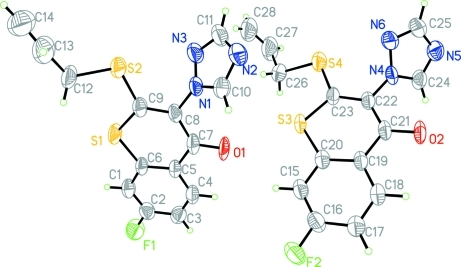
The molecular structure of the title compound with the atom-numbering scheme. Displacement ellipsoids are drawn at the 50% probability level.

**Fig. 2.**
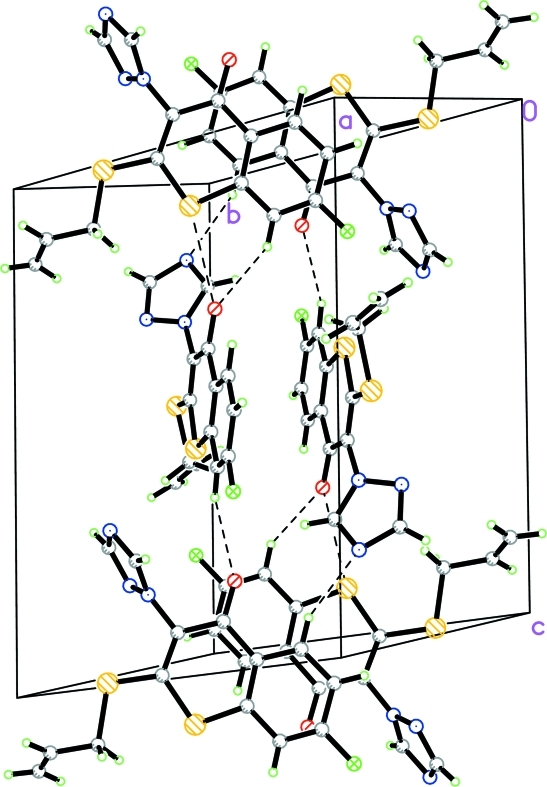
A packing diagram of the title compound.

## supplementary crystallographic information

### Comment

The title compound, C_14_H_10_FN_3_OS_2_, is a new molecule which has a potential use as antifungal. We herein report its crystal structure. The bond lengths and angles are within normal ranges (Allen *et al.*, 1987). The asymmetric unit contains two independent molecules. They differ in the relative orientations of the triazole and allylsulfanyl groups with respect to the planar thiochromen-4-one frameworks. The dihedral angles N3—N1—C8—C7 and the corresponding N6—N4—C22—C21 are 128.2 (5)° and -120.9 (5)° while the dihedral angles C26—S4—C23—S3 and C12—S2—C9—S1 are -17.4 (4)° and 16.4 (4)°. The two-ring system is essentially planar in each molecule. The dihedral angles between the mean planes of the benzene rings and of the C_5_S rings are 2.7 (2) and 2.9 (2)°. In the crystal structure, intermolecular C—H···O and C—H···N hydrogen bonds link the molecules in a stacked arrangement along the *a* axis.

### Experimental

CS_2 _(2.0 g, 26.3 mmol) was dropwise added to a solution of 1-(2,4- difluorophenyl)-2-(1*H*-1,2,4-triazol-1-yl)ethanone (5 g, 22.4 mmol) in DMSO (20 ml) containing NaOH (1.8 g, 45 mmol). The yellow solution was stirred for about 2 h at room temperature. Then allyl chloride (1.7 g, 22.4 mmol) was dropwise added to the intermediate. After 3 h, the solution was poured into water (50 ml). The crystalline product was isolated by filtration, washed with water (300 ml). The crystals were obtained by dissolving the title compound in acetone (20 ml) and evaporating acetone slowly at room temperature for about 7 d.

### Refinement

The H atoms were positioned geometrically with C—H = 0.93 Å for aromatic H atoms and C—H = 0.97 Å for methylene H atoms, and constrained to ride on their parent atoms, with *U*_iso_(H) = 1.2*U*eq(C).

### Crystal data

**Table:**

C_14_H_10_FN_3_OS_2_	*Z* = 4
*M**_r_* = 319.39	*F*(000) = 656
Triclinic, *P*1	*D*_x_ = 1.514 Mg m^−^^3^
Hall symbol: -P 1	Melting point: 385 K
*a* = 8.1730 (16) Å	Mo *K*α radiation, λ = 0.71073 Å
*b* = 11.646 (2) Å	Cell parameters from 25 reflections
*c* = 15.124 (3) Å	θ = 9–12°
α = 82.43 (3)°	µ = 0.39 mm^−^^1^
β = 83.98 (3)°	*T* = 293 K
γ = 80.14 (3)°	Block, yellow
*V* = 1400.9 (5) Å^3^	0.20 × 0.10 × 0.10 mm

### Data collection

**Table:**

Enraf–Nonius CAD-4 diffractometer	3087 reflections with *I* > 2σ(*I*)
Radiation source: fine-focus sealed tube	*R*_int_ = 0.037
graphite	θ_max_ = 25.5°, θ_min_ = 1.4°
ω/2θ scans	*h* = 0→9
Absorption correction: ψ scan (North *et al.*, 1968)	*k* = −13→14
*T*_min_ = 0.926, *T*_max_ = 0.962	*l* = −18→18
5541 measured reflections	3 standard reflections every 200 reflections
5149 independent reflections	intensity decay: 1%

### Refinement

**Table:**

Refinement on *F*^2^	Primary atom site location: structure-invariant direct methods
Least-squares matrix: full	Secondary atom site location: difference Fourier map
*R*[*F*^2^ > 2σ(*F*^2^)] = 0.069	Hydrogen site location: inferred from neighbouring sites
*wR*(*F*^2^) = 0.176	H-atom parameters constrained
*S* = 1.01	*w* = 1/[σ^2^(*F*_o_^2^) + (0.080*P*)^2^ + 1.*P*] where *P* = (*F*_o_^2^ + 2*F*_c_^2^)/3
5149 reflections	(Δ/σ)_max_ = 0.001
379 parameters	Δρ_max_ = 0.52 e Å^−^^3^
1 restraint	Δρ_min_ = −0.40 e Å^−^^3^

### Special details

**Table:**

Geometry. All e.s.d.'s (except the e.s.d. in the dihedral angle between two l.s. planes) are estimated using the full covariance matrix. The cell e.s.d.'s are taken into account individually in the estimation of e.s.d.'s in distances, angles and torsion angles; correlations between e.s.d.'s in cell parameters are only used when they are defined by crystal symmetry. An approximate (isotropic) treatment of cell e.s.d.'s is used for estimating e.s.d.'s involving l.s. planes.
Refinement. Refinement of *F*^2^ against ALL reflections. The weighted *R*-factor *wR* and goodness of fit *S* are based on *F*^2^, conventional *R*-factors *R* are based on *F*, with *F* set to zero for negative *F*^2^. The threshold expression of *F*^2^ > σ(*F*^2^) is used only for calculating *R*-factors(gt) *etc*. and is not relevant to the choice of reflections for refinement. *R*-factors based on *F*^2^ are statistically about twice as large as those based on *F*, and *R*- factors based on ALL data will be even larger.

### Fractional atomic coordinates and isotropic or equivalent isotropic displacement parameters (Å^2^)

**Table:**

	*x*	*y*	*z*	*U*_iso_*/*U*_eq_	
S1	0.44048 (18)	0.30108 (12)	0.42901 (7)	0.0563 (4)	
O1	0.3291 (5)	0.4374 (3)	0.69021 (19)	0.0718 (11)	
F1	0.0540 (4)	0.6601 (3)	0.3288 (2)	0.0787 (10)	
N1	0.5146 (5)	0.2210 (3)	0.6888 (2)	0.0472 (10)	
C1	0.2380 (6)	0.4976 (4)	0.3820 (3)	0.0511 (12)	
H1A	0.2597	0.4748	0.3247	0.061*	
S2	0.64594 (19)	0.10674 (12)	0.52776 (8)	0.0604 (4)	
N2	0.6087 (6)	0.1689 (4)	0.8194 (3)	0.0640 (12)	
C2	0.1322 (7)	0.5970 (5)	0.3969 (3)	0.0538 (13)	
C3	0.1006 (6)	0.6363 (4)	0.4802 (3)	0.0552 (13)	
H3A	0.0285	0.7057	0.4886	0.066*	
N3	0.4931 (6)	0.1073 (4)	0.7111 (2)	0.0610 (12)	
C4	0.1774 (6)	0.5709 (4)	0.5491 (3)	0.0526 (13)	
H4A	0.1579	0.5967	0.6054	0.063*	
C5	0.2847 (6)	0.4662 (4)	0.5386 (3)	0.0414 (11)	
C6	0.3135 (6)	0.4303 (4)	0.4532 (3)	0.0419 (11)	
C7	0.3553 (6)	0.3979 (4)	0.6171 (3)	0.0495 (12)	
C8	0.4592 (6)	0.2856 (4)	0.6084 (3)	0.0428 (11)	
C9	0.5093 (6)	0.2390 (4)	0.5298 (3)	0.0457 (11)	
C10	0.5830 (7)	0.2549 (5)	0.7553 (3)	0.0563 (14)	
H10A	0.6088	0.3295	0.7559	0.068*	
C11	0.5504 (8)	0.0820 (5)	0.7890 (3)	0.0659 (16)	
H11A	0.5510	0.0085	0.8218	0.079*	
C12	0.7155 (8)	0.1027 (5)	0.4101 (3)	0.0734 (18)	
H12A	0.6357	0.0727	0.3799	0.088*	
H12B	0.7267	0.1810	0.3817	0.088*	
C13	0.8773 (11)	0.0255 (7)	0.4051 (4)	0.108 (3)	
H13A	0.9647	0.0519	0.4269	0.129*	
C14	0.9121 (11)	−0.0722 (6)	0.3748 (4)	0.112 (3)	
H14A	0.8299	−0.1031	0.3520	0.134*	
H14B	1.0201	−0.1134	0.3751	0.134*	
S3	0.24689 (18)	0.41381 (11)	0.89646 (7)	0.0544 (4)	
S4	0.1326 (2)	0.20977 (13)	1.00091 (8)	0.0656 (4)	
F2	0.4001 (4)	0.8034 (3)	0.78292 (18)	0.0770 (10)	
N4	0.1606 (5)	0.3240 (3)	1.1599 (2)	0.0478 (10)	
N5	0.0575 (6)	0.2723 (4)	1.2953 (3)	0.0605 (12)	
N6	0.2392 (6)	0.2129 (4)	1.1815 (3)	0.0635 (13)	
O2	0.2657 (5)	0.5303 (3)	1.15925 (19)	0.0618 (10)	
C15	0.3312 (6)	0.6232 (4)	0.8431 (3)	0.0516 (13)	
H15A	0.3294	0.6027	0.7859	0.062*	
C16	0.3687 (7)	0.7272 (5)	0.8545 (3)	0.0553 (13)	
C17	0.3756 (7)	0.7615 (5)	0.9382 (3)	0.0568 (13)	
H17A	0.4038	0.8339	0.9444	0.068*	
C18	0.3395 (6)	0.6851 (4)	1.0112 (3)	0.0518 (13)	
H18A	0.3440	0.7063	1.0680	0.062*	
C19	0.2964 (6)	0.5765 (4)	1.0038 (3)	0.0417 (11)	
C20	0.2946 (6)	0.5460 (4)	0.9182 (3)	0.0431 (11)	
C21	0.2567 (6)	0.4993 (4)	1.0857 (3)	0.0428 (11)	
C22	0.2014 (6)	0.3908 (4)	1.0784 (3)	0.0416 (11)	
C23	0.1920 (6)	0.3458 (4)	1.0007 (3)	0.0463 (12)	
C24	0.0550 (6)	0.3569 (5)	1.2292 (3)	0.0528 (13)	
H24A	−0.0114	0.4301	1.2301	0.063*	
C25	0.1744 (8)	0.1885 (5)	1.2630 (3)	0.0685 (16)	
H25A	0.2072	0.1171	1.2969	0.082*	
C26	0.0874 (8)	0.2065 (5)	0.8870 (3)	0.0665 (16)	
H26A	0.0070	0.2745	0.8682	0.080*	
H26B	0.1882	0.2071	0.8470	0.080*	
C27	0.0185 (10)	0.0971 (6)	0.8850 (4)	0.094 (2)	
H27A	−0.0860	0.0931	0.9150	0.113*	
C28	0.0857 (11)	0.0102 (6)	0.8476 (4)	0.105 (3)	
H28C	0.1904	0.0097	0.8166	0.126*	
H28A	0.0315	−0.0543	0.8504	0.126*	

### Atomic displacement parameters (Å^2^)

**Table:**

	*U*^11^	*U*^22^	*U*^33^	*U*^12^	*U*^13^	*U*^23^
S1	0.0737 (10)	0.0671 (9)	0.0274 (6)	0.0032 (7)	−0.0070 (5)	−0.0204 (5)
O1	0.108 (3)	0.080 (3)	0.0250 (16)	0.005 (2)	−0.0030 (17)	−0.0291 (16)
F1	0.091 (3)	0.081 (2)	0.0559 (18)	0.0140 (19)	−0.0192 (17)	−0.0051 (16)
N1	0.067 (3)	0.052 (2)	0.0259 (18)	−0.015 (2)	−0.0017 (17)	−0.0117 (16)
C1	0.056 (3)	0.068 (3)	0.033 (2)	−0.012 (3)	−0.004 (2)	−0.014 (2)
S2	0.0812 (10)	0.0641 (9)	0.0325 (6)	0.0045 (7)	−0.0051 (6)	−0.0139 (6)
N2	0.086 (4)	0.068 (3)	0.040 (2)	−0.010 (3)	−0.017 (2)	−0.007 (2)
C2	0.060 (3)	0.059 (3)	0.042 (3)	−0.009 (3)	−0.007 (2)	−0.003 (2)
C3	0.054 (3)	0.055 (3)	0.054 (3)	−0.002 (3)	0.001 (2)	−0.013 (2)
N3	0.095 (4)	0.059 (3)	0.034 (2)	−0.022 (2)	−0.005 (2)	−0.0076 (18)
C4	0.060 (3)	0.063 (3)	0.035 (2)	−0.009 (3)	0.005 (2)	−0.015 (2)
C5	0.043 (3)	0.054 (3)	0.030 (2)	−0.012 (2)	0.0027 (19)	−0.0129 (19)
C6	0.047 (3)	0.054 (3)	0.028 (2)	−0.012 (2)	−0.0005 (19)	−0.0150 (19)
C7	0.056 (3)	0.068 (3)	0.029 (2)	−0.017 (3)	0.003 (2)	−0.018 (2)
C8	0.055 (3)	0.052 (3)	0.025 (2)	−0.017 (2)	−0.0035 (19)	−0.0091 (19)
C9	0.051 (3)	0.058 (3)	0.033 (2)	−0.016 (2)	−0.002 (2)	−0.014 (2)
C10	0.079 (4)	0.056 (3)	0.038 (3)	−0.011 (3)	−0.014 (2)	−0.016 (2)
C11	0.099 (5)	0.066 (4)	0.034 (3)	−0.019 (3)	−0.003 (3)	−0.006 (2)
C12	0.089 (4)	0.084 (4)	0.034 (3)	0.028 (3)	−0.001 (3)	−0.018 (3)
C13	0.143 (7)	0.116 (6)	0.047 (4)	0.031 (5)	−0.007 (4)	−0.013 (4)
C14	0.152 (8)	0.096 (6)	0.077 (5)	0.025 (5)	−0.022 (5)	−0.018 (4)
S3	0.0840 (10)	0.0579 (8)	0.0237 (5)	−0.0125 (7)	−0.0015 (5)	−0.0138 (5)
S4	0.0998 (12)	0.0617 (9)	0.0420 (7)	−0.0247 (8)	−0.0080 (7)	−0.0136 (6)
F2	0.113 (3)	0.081 (2)	0.0412 (16)	−0.039 (2)	−0.0052 (16)	0.0077 (15)
N4	0.062 (3)	0.057 (3)	0.0225 (18)	−0.005 (2)	−0.0003 (17)	−0.0076 (16)
N5	0.076 (3)	0.064 (3)	0.039 (2)	−0.014 (2)	0.013 (2)	−0.008 (2)
N6	0.089 (4)	0.056 (3)	0.036 (2)	0.001 (2)	0.010 (2)	−0.0021 (18)
O2	0.098 (3)	0.071 (2)	0.0242 (16)	−0.025 (2)	−0.0040 (16)	−0.0166 (15)
C15	0.065 (3)	0.061 (3)	0.031 (2)	−0.013 (3)	−0.006 (2)	−0.006 (2)
C16	0.064 (4)	0.065 (3)	0.036 (3)	−0.016 (3)	0.000 (2)	0.001 (2)
C17	0.075 (4)	0.059 (3)	0.041 (3)	−0.020 (3)	−0.005 (2)	−0.008 (2)
C18	0.065 (3)	0.060 (3)	0.033 (2)	−0.011 (3)	−0.008 (2)	−0.011 (2)
C19	0.045 (3)	0.051 (3)	0.028 (2)	0.000 (2)	−0.0042 (18)	−0.0087 (19)
C20	0.044 (3)	0.052 (3)	0.033 (2)	−0.004 (2)	−0.0044 (19)	−0.0087 (19)
C21	0.047 (3)	0.056 (3)	0.025 (2)	−0.004 (2)	−0.0011 (18)	−0.0099 (19)
C22	0.046 (3)	0.049 (3)	0.029 (2)	−0.005 (2)	−0.0012 (19)	−0.0078 (19)
C23	0.056 (3)	0.052 (3)	0.030 (2)	−0.002 (2)	−0.002 (2)	−0.012 (2)
C24	0.062 (3)	0.061 (3)	0.037 (3)	−0.013 (3)	0.008 (2)	−0.015 (2)
C25	0.098 (5)	0.064 (4)	0.036 (3)	−0.004 (3)	0.004 (3)	0.002 (2)
C26	0.098 (5)	0.057 (3)	0.048 (3)	−0.011 (3)	−0.011 (3)	−0.018 (2)
C27	0.131 (6)	0.103 (6)	0.060 (4)	−0.042 (5)	−0.009 (4)	−0.028 (4)
C28	0.166 (8)	0.092 (5)	0.066 (4)	−0.037 (5)	0.001 (4)	−0.032 (4)

### Geometric parameters (Å, °)

**Table:**

S1—C9	1.706 (5)	S3—C23	1.720 (4)
S1—C6	1.734 (5)	S3—C20	1.731 (5)
O1—C7	1.237 (5)	S4—C23	1.734 (5)
F1—C2	1.337 (5)	S4—C26	1.806 (5)
N1—C10	1.334 (5)	F2—C16	1.339 (5)
N1—N3	1.359 (5)	N4—C24	1.339 (5)
N1—C8	1.416 (5)	N4—N6	1.357 (5)
C1—C2	1.350 (7)	N4—C22	1.409 (5)
C1—C6	1.380 (6)	N5—C24	1.307 (6)
C1—H1A	0.9300	N5—C25	1.347 (6)
S2—C9	1.743 (5)	N6—C25	1.305 (6)
S2—C12	1.814 (5)	O2—C21	1.227 (5)
N2—C10	1.305 (6)	C15—C16	1.335 (7)
N2—C11	1.340 (7)	C15—C20	1.393 (6)
C2—C3	1.381 (6)	C15—H15A	0.9300
C3—C4	1.350 (7)	C16—C17	1.387 (6)
C3—H3A	0.9300	C17—C18	1.364 (6)
N3—C11	1.293 (6)	C17—H17A	0.9300
C4—C5	1.392 (6)	C18—C19	1.391 (6)
C4—H4A	0.9300	C18—H18A	0.9300
C5—C6	1.393 (5)	C19—C20	1.389 (6)
C5—C7	1.454 (6)	C19—C21	1.473 (6)
C7—C8	1.446 (7)	C21—C22	1.435 (6)
C8—C9	1.367 (6)	C22—C23	1.362 (6)
C10—H10A	0.9300	C24—H24A	0.9300
C11—H11A	0.9300	C25—H25A	0.9300
C12—C13	1.467 (9)	C26—C27	1.484 (8)
C12—H12A	0.9700	C26—H26A	0.9700
C12—H12B	0.9700	C26—H26B	0.9700
C13—C14	1.259 (7)	C27—C28	1.241 (9)
C13—H13A	0.9300	C27—H27A	0.9300
C14—H14A	0.9300	C28—H28C	0.9300
C14—H14B	0.9300	C28—H28A	0.9300
			
C9—S1—C6	104.0 (2)	C23—S3—C20	103.9 (2)
C10—N1—N3	108.5 (4)	C23—S4—C26	104.5 (2)
C10—N1—C8	130.2 (4)	C24—N4—N6	109.1 (4)
N3—N1—C8	121.2 (4)	C24—N4—C22	128.8 (4)
C2—C1—C6	118.9 (4)	N6—N4—C22	121.9 (4)
C2—C1—H1A	120.5	C24—N5—C25	101.9 (4)
C6—C1—H1A	120.5	C25—N6—N4	101.7 (4)
C9—S2—C12	103.8 (2)	C16—C15—C20	119.0 (4)
C10—N2—C11	102.7 (4)	C16—C15—H15A	120.5
F1—C2—C1	119.2 (4)	C20—C15—H15A	120.5
F1—C2—C3	118.3 (5)	C15—C16—F2	119.7 (4)
C1—C2—C3	122.5 (5)	C15—C16—C17	122.9 (5)
C4—C3—C2	118.0 (5)	F2—C16—C17	117.5 (5)
C4—C3—H3A	121.0	C18—C17—C16	117.5 (5)
C2—C3—H3A	121.0	C18—C17—H17A	121.2
C11—N3—N1	102.7 (4)	C16—C17—H17A	121.2
C3—C4—C5	122.2 (4)	C17—C18—C19	122.4 (4)
C3—C4—H4A	118.9	C17—C18—H18A	118.8
C5—C4—H4A	118.9	C19—C18—H18A	118.8
C4—C5—C6	117.8 (4)	C20—C19—C18	117.6 (4)
C4—C5—C7	118.7 (4)	C20—C19—C21	123.2 (4)
C6—C5—C7	123.5 (4)	C18—C19—C21	119.3 (4)
C1—C6—C5	120.5 (4)	C19—C20—C15	120.7 (4)
C1—C6—S1	115.9 (3)	C19—C20—S3	123.8 (4)
C5—C6—S1	123.6 (4)	C15—C20—S3	115.5 (3)
O1—C7—C8	120.6 (4)	O2—C21—C22	120.8 (4)
O1—C7—C5	119.9 (5)	O2—C21—C19	119.8 (4)
C8—C7—C5	119.5 (4)	C22—C21—C19	119.4 (4)
C9—C8—N1	118.4 (4)	C23—C22—N4	118.3 (4)
C9—C8—C7	125.4 (4)	C23—C22—C21	125.8 (4)
N1—C8—C7	116.2 (4)	N4—C22—C21	115.8 (4)
C8—C9—S1	123.7 (4)	C22—C23—S3	123.6 (4)
C8—C9—S2	120.8 (4)	C22—C23—S4	121.4 (4)
S1—C9—S2	115.5 (2)	S3—C23—S4	114.9 (2)
N2—C10—N1	110.6 (5)	N5—C24—N4	111.0 (5)
N2—C10—H10A	124.7	N5—C24—H24A	124.5
N1—C10—H10A	124.7	N4—C24—H24A	124.5
N3—C11—N2	115.5 (5)	N6—C25—N5	116.2 (5)
N3—C11—H11A	122.3	N6—C25—H25A	121.9
N2—C11—H11A	122.3	N5—C25—H25A	121.9
C13—C12—S2	107.3 (4)	C27—C26—S4	107.0 (4)
C13—C12—H12A	110.3	C27—C26—H26A	110.3
S2—C12—H12A	110.3	S4—C26—H26A	110.3
C13—C12—H12B	110.3	C27—C26—H26B	110.3
S2—C12—H12B	110.3	S4—C26—H26B	110.3
H12A—C12—H12B	108.5	H26A—C26—H26B	108.6
C14—C13—C12	127.8 (9)	C28—C27—C26	126.9 (8)
C14—C13—H13A	116.1	C28—C27—H27A	116.6
C12—C13—H13A	116.1	C26—C27—H27A	116.6
C13—C14—H14A	120.0	C27—C28—H28C	120.0
C13—C14—H14B	120.0	C27—C28—H28A	120.0
H14A—C14—H14B	120.0	H28C—C28—H28A	120.0
			
C6—C1—C2—F1	−177.5 (4)	C24—N4—N6—C25	−0.5 (6)
C6—C1—C2—C3	2.2 (8)	C22—N4—N6—C25	175.2 (5)
F1—C2—C3—C4	178.7 (5)	C20—C15—C16—F2	−178.4 (4)
C1—C2—C3—C4	−1.0 (8)	C20—C15—C16—C17	1.0 (8)
C10—N1—N3—C11	−0.1 (6)	C15—C16—C17—C18	−1.0 (8)
C8—N1—N3—C11	−176.8 (5)	F2—C16—C17—C18	178.4 (5)
C2—C3—C4—C5	−0.5 (8)	C16—C17—C18—C19	−0.3 (8)
C3—C4—C5—C6	0.9 (7)	C17—C18—C19—C20	1.5 (7)
C3—C4—C5—C7	−176.7 (5)	C17—C18—C19—C21	−179.1 (5)
C2—C1—C6—C5	−1.9 (7)	C18—C19—C20—C15	−1.5 (7)
C2—C1—C6—S1	177.3 (4)	C21—C19—C20—C15	179.1 (4)
C4—C5—C6—C1	0.4 (7)	C18—C19—C20—S3	179.1 (4)
C7—C5—C6—C1	177.8 (4)	C21—C19—C20—S3	−0.3 (7)
C4—C5—C6—S1	−178.8 (4)	C16—C15—C20—C19	0.3 (7)
C7—C5—C6—S1	−1.3 (7)	C16—C15—C20—S3	179.8 (4)
C9—S1—C6—C1	−178.8 (4)	C23—S3—C20—C19	4.4 (5)
C9—S1—C6—C5	0.4 (5)	C23—S3—C20—C15	−175.1 (4)
C4—C5—C7—O1	−5.2 (7)	C20—C19—C21—O2	177.7 (4)
C6—C5—C7—O1	177.4 (5)	C18—C19—C21—O2	−1.7 (7)
C4—C5—C7—C8	176.4 (4)	C20—C19—C21—C22	−4.7 (7)
C6—C5—C7—C8	−1.0 (7)	C18—C19—C21—C22	175.9 (4)
C10—N1—C8—C9	131.2 (5)	C24—N4—C22—C23	−128.9 (5)
N3—N1—C8—C9	−52.9 (6)	N6—N4—C22—C23	56.5 (6)
C10—N1—C8—C7	−47.7 (7)	C24—N4—C22—C21	53.8 (7)
N3—N1—C8—C7	128.2 (5)	N6—N4—C22—C21	−120.9 (5)
O1—C7—C8—C9	−173.4 (5)	O2—C21—C22—C23	−177.6 (5)
C5—C7—C8—C9	5.1 (7)	C19—C21—C22—C23	4.9 (7)
O1—C7—C8—N1	5.5 (7)	O2—C21—C22—N4	−0.5 (7)
C5—C7—C8—N1	−176.1 (4)	C19—C21—C22—N4	−178.0 (4)
N1—C8—C9—S1	174.9 (3)	N4—C22—C23—S3	−176.9 (3)
C7—C8—C9—S1	−6.3 (7)	C21—C22—C23—S3	0.1 (7)
N1—C8—C9—S2	−3.1 (6)	N4—C22—C23—S4	−0.3 (6)
C7—C8—C9—S2	175.8 (4)	C21—C22—C23—S4	176.8 (4)
C6—S1—C9—C8	3.3 (5)	C20—S3—C23—C22	−4.3 (5)
C6—S1—C9—S2	−178.7 (3)	C20—S3—C23—S4	178.8 (3)
C12—S2—C9—C8	−165.5 (4)	C26—S4—C23—C22	165.7 (4)
C12—S2—C9—S1	16.4 (4)	C26—S4—C23—S3	−17.4 (4)
C11—N2—C10—N1	−0.9 (6)	C25—N5—C24—N4	2.0 (6)
N3—N1—C10—N2	0.6 (6)	N6—N4—C24—N5	−1.1 (6)
C8—N1—C10—N2	176.9 (5)	C22—N4—C24—N5	−176.3 (4)
N1—N3—C11—N2	−0.5 (7)	N4—N6—C25—N5	1.9 (7)
C10—N2—C11—N3	0.9 (7)	C24—N5—C25—N6	−2.5 (7)
C9—S2—C12—C13	156.2 (5)	C23—S4—C26—C27	−174.1 (5)
S2—C12—C13—C14	113.6 (7)	S4—C26—C27—C28	−111.7 (7)

### Hydrogen-bond geometry (Å, °)

**Table:**

*D*—H···*A*	*D*—H	H···*A*	*D*···*A*	*D*—H···*A*
C1—H1A···O2^i^	0.93	2.50	3.328 (5)	149
C4—H4A···N5^ii^	0.93	2.61	3.397 (7)	143
C15—H15A···O1	0.93	2.56	3.372 (6)	147
C18—H18A···N2^iii^	0.93	2.48	3.355 (7)	157

Symmetry codes: (i) *x*, *y*, *z*−1; (ii) −*x*, −*y*+1, −*z*+2; (iii) −*x*+1, −*y*+1, −*z*+2.
